# Leveraging comparative phylogenetics for evolutionary medicine: applications to comparative oncology

**DOI:** 10.1093/emph/eoaf039

**Published:** 2025-12-24

**Authors:** Walker J Compton Mellon, Beckett Sterner, J Arvid Ågren, Orsolya Vincze, Matthew Marx, Stefania Kapsetaki, Ping-Han Huang, Bryan Yavari, Hunter W McCollum, B Natterson-Horowitz, Hannah Human, Cristina Baciu, Harley Richker, Diego Mallo, Carlo C Maley, Luke Harmon, Zachary T Compton

**Affiliations:** Arizona Cancer Evolution Center, The Biodesign Institute, Tempe, AZ, USA; University of Arizona College of Medicine, Phoenix, AZ, USA; School of Life Sciences, Arizona State University, Tempe, AZ, USA; Lerner Research Institute, Cleveland Clinic Foundation, Cleveland, OH, USA; Institute of Aquatic Ecology, Centre for Ecological Research, Debrecen, Hungary; Evolutionary Ecology Group, Hungarian Department of Biology and Ecology, Babeş-Bolyai University, Cluj-Napoca, Romania; Arizona Cancer Evolution Center, The Biodesign Institute, Tempe, AZ, USA; Arizona Cancer Evolution Center, The Biodesign Institute, Tempe, AZ, USA; School of Health Sciences, Department of Pharmacology, Frederick University, Nicosia, Cyprus; Hellenic Open University, Patras, Greece; School of Mathematical and Statistical Sciences, Arizona State University, Tempe, AZ, USA; Arizona Cancer Evolution Center, The Biodesign Institute, Tempe, AZ, USA; University of Arizona College of Medicine, Phoenix, AZ, USA; Indiana University School of Medicine, Indianapolis, IN, USA; Department of Human Evolutionary Biology, Harvard University, Cambridge, MA, USA; Arizona Cancer Evolution Center, The Biodesign Institute, Tempe, AZ, USA; Arizona Cancer Evolution Center, The Biodesign Institute, Tempe, AZ, USA; Arizona Cancer Evolution Center, The Biodesign Institute, Tempe, AZ, USA; Arizona Cancer Evolution Center, The Biodesign Institute, Tempe, AZ, USA; Arizona Cancer Evolution Center, The Biodesign Institute, Tempe, AZ, USA; University of Arizona College of Medicine, Phoenix, AZ, USA; Department of Biological Sciences, University of Idaho, Moscow, Russia; Arizona Cancer Evolution Center, The Biodesign Institute, Tempe, AZ, USA; University of Arizona Cancer Center, Tucson, AZ, USA; University of Arizona College of Medicine, Tucson, AZ, USA

**Keywords:** evolutionary medicine, evolutionary biology, phylogenetics, bioinformatics, statistical modeling

## Abstract

Comparative phylogenetics provides a wealth of computational tools to understand evolutionary processes and their outcomes. Advances in these methodologies have occurred in parallel with a surge in cross-species genomic and phenotypic data. To date, however, the majority of published studies have focused on classical questions in evolutionary biology, such as speciation and the ecological drivers of trait evolution. Here, we argue that evolutionary medicine in general, and our understanding of the origin and diversification of disease traits in particular, would be greatly expanded by a wider integration of phylogenetic comparative methods (PCMs). We use comparative oncology—the study of cancer across the tree of life—as an example to demonstrate the power of the approach and show that implementing PCMs can highlight the mode and tempo of the evolutionary changes in intrinsic, species-level disease vulnerabilities.

## BACKGROUND AND OBJECTIVES

Evolutionary medicine seeks to understand the ultimate origin and persistence of human disease within the context of the species’ evolutionary history and that of their relatives [1–3]. A central explanatory concept in the field has been the idea of evolutionary mismatch [4, 5], which hypothesizes that contemporary maladies are often rooted in the dramatic shift in environment humans have undergone on a timescale that natural selection cannot meaningfully act upon. In the last decade, human diseases have been explored through this lens, including autoimmune disorders, diabetes, heart disease, and psychiatric disorders [1, 2, 6–14].

The evolutionary mismatch framework has been incredibly productive in generating hypotheses to explain several human maladies and our bodies’ response to them. From psychiatric conditions to autoimmune disease, evolutionary mismatch has worked to not only explain disease but also offer interventions [9, 12, 13, 15]. To date, however, the majority of research in evolutionary medicine has focused on humans (*Homo sapiens)* and our immediate ancestors, with relatively little attention paid to the context of broader phylogeny. For example, early attempts to explain the variation in cancer risk across taxa heavily relied on a life history framework of disease risk among humans and our closest relatives [16–20]. Given the diverse ecological backgrounds in which species evolve, different strategies will dictate the amount of energetic investment into somatic maintenance and other disease relevant domains. Moreover, the application of evolutionary trait models can drive deeper testing of the hypotheses developed from a life history framework of disease vulnerability. Life history theory has provided a powerful toolkit for understanding the evolution of reproductive and survival strategies across different species. More recently, this framework has been applied to studies of cross-species cancer prevalence, and a growing body of research suggests that cancer susceptibility may be linked to reduced energetic investment in somatic maintenance, favoring a high-volume reproductive output instead, as suggested by fast life history theory [16, 21–23]. Yet, there is more to be learned about human maladies when we expand our focal point to examine disease across deeper evolutionary history. In recent years, the integration of phylogenetic comparative methods (henceforth, PCMs), the study of evolutionary relationships among species, has provided such avenues for investigating the evolutionary origins and diversification of disease risk.

The comparative method is a cornerstone of evolutionary biology [24]. However, it was not until the 1980s that the advancement of PCMs made significant headway, due in equal parts to the development of robust statistical approaches and confidence in DNA-based phylogenies [25–28]. (‘The comparative method of 1950 was indistinguishable from the comparative method of 350 BC,’ as Ridley 1983, p. 6 put it [29].) By now, the rigor of statistical and computational techniques within comparative phylogenetics has come to rival that of population genetics. The continuous growth in computational methods and the accumulation of vast amounts of genomic and phenotypic data across a wide range of species has made PCMs increasingly popular. While attempts to apply PCMs to questions in evolutionary medicine have increased, efforts to outline ‘best practices’ for PCMs in evolutionary medicine have been sparse [30, 31].

In this review, we argue that the integration of PCMs in evolutionary medicine can greatly enhance our understanding of disease risk, which may inform novel strategies for prevention and treatment of disease. Drawing on recent advances in PCMs and evolutionary medicine, we highlight the key benefits of this integrative approach and provide examples of its applications in the study of comparative oncology—the study of cancer across the tree of life [32–35]. We begin by outlining some of the basics of the comparative method and then apply it to a previously published dataset of cross-species cancer prevalence. We end by discussing the challenges and limitations of using PCMs in the context of human health, and provide a roadmap for its applications in broader evolutionary medicine topics.

### A primer on PCMs

PCMs are a suite of statistical and computational tools that utilize phylogenetic information to study how species evolve and how their traits are related. These methods use evolutionary trees (phylogenies) to provide an evolutionary framework for these analyses [36]. PCMs can be placed into two main categories: those that estimate the tempo and mode of evolution, and those that examine relationships between traits and the environment [37]. One important focus is reconstructing traits’ evolutionary trajectories using various evolutionary models and incorporating that history into subsequent statistical analysis. These methods often build upon familiar statistical approaches, such as regression, t-test, analysis of variance (ANOVA), and correlation, by incorporating phylogenetic information to account for the non-independence of species due to shared ancestry [38]. This framework has been integrated into a wide range of statistical methods, leaving researchers with a diverse set of tools that, at times, can be challenging to choose from.

### Models of phenotype evolution

The foundation of modern models of continuous trait evolution rests with Brownian motion (discrete traits—traits with distinct states—are typically modeled with a model called the Markov k-state, or Mk, model). Models of Brownian motion originally developed from attempts to explain the outcome of processes that are seemingly the product of random interactions, specifically the random movement of particles suspended in a medium (fluid or gas). First described while observing the ‘irregular motion’ of coal dust on the surface of alcohol by Jan Ingenhousz, the Brownian motion is actually named after the work of botanist Robert Brown, some decades later. Brownian motion is the basis of a large family of mathematical models that are popular due to their mathematical tractability and are suitable for modeling phenomena where small stochastic displacements add up to approximate Gaussian fluctuations. In comparative phylogenetics, data that has changed over time is plentiful, in the form of the average values of continuous evolutionary traits within populations or species. Many such traits are well-fit by Brownian motion. Naturally, early phylogenetic studies and research applied the Brownian model to the divergence of traits over time rather successfully. So it follows that this is often the basis of the null hypothesis in models of phylogenetic context [36]. Although one possible mechanism for random change fitting a Brownian model is neutral ‘genetic drift’, there are other possible mechanisms for Brownian motion involving selection [39, 40]. In cases where Brownian motion is the most suitable model for a trait, it is characterized by a ‘random walk,’ signifying a lack of directionality or identifiable trends in variation. Given that Brownian motion is commonly a well-supported model, it provides a useful null hypothesis in investigating alternative modes of phenotypic evolutionary change [41]. Building on the foundational model of Brownian motion, alternative evolutionary models have been developed over the past decades that are now able to incorporate various evolutionary forces, such as directional selection, adaptive radiation, and evolutionary constraints, to better fit empirical data [38, 42–45]. Crucially, advances in computational modeling have also expanded the range of questions we can ask about the evolutionary forces that drive the extensive variation observed in species’ traits.

A prominent extension of Brownian motion, used widely in mathematical and physical frameworks, is the Ornstein–Uhlenbeck (OU) model, which describes trait evolution that reverts toward an optimal or mean value. George Eugene Uhlenbeck and Leonard Ornstein devised the model in 1930 to describe the velocity of a Brownian particle undergoing friction (resistance to change) [46]. OU has broad applications in physics, finance, and evolutionary biology. The OU model of evolution was adapted from models to explain random movements in a statistical framework to describe the evolution of continuous traits in a population over time [47]. Evolutionary biologists commonly interpret this model as incorporating both stabilizing selection and genetic drift to explain how a trait may evolve in response to stochastic displacements around a center, e.g. the fitness optimum [48]. An OU model includes a stochastic component, similar to Brownian motion, while the deterministic component describes the pull toward an optimal value due to stabilizing selection or any other restraining force. ​​.

Beyond the OU model, other evolutionary models further expand on Brownian motion to capture various aspects of evolution. For instance, the rate trend model is variation of the Brownian motion model with linear trends in traits or step variance (i.e. how fast a trait spreads around a mean) that describe the rate of change in continuous traits over time [43, 49]. These rates can be either positive (indicating increasing rates over time) or negative (indicating decreasing rates over time). This model can explain how evolutionary pressures and environmental changes influence or inhibit rates of trait change.

Another early approach to stepping beyond Brownian motion was suggested by Mark Pagel in 1999 [50]. While the originally suggested approach was designed for discrete traits, the author later pointed out that it applies equally well to continuous traits, and has been used extensively ever since. In traditional explicit evolutionary models, such as Brownian motion and its extensions, trait evolution is modeled by explicitly defining and adjusting parameters that represent various evolutionary processes driving trait change. Pagel’s frameworks of tree transformations differ from the traditional explicit evolutionary models discussed above. Instead of directly modeling how traits evolve, Pagel’s model’s (λ, δ, and κ) modify the structure of the phylogeny by adjusting branch lengths or node positions, which changes how similar species’ are expected to be across the tree.

Pagel’s λ values range from 0, meaning species’ trait values are statistically independent of the phylogeny, to 1, indicating trait values resulting from Brownian motion. Intermediate values indicate partial phylogenetic influence. Pagel’s λ describes how much shared ancestry explains trait variation across species, also known as phylogenetic signal [51]. The δ transformation adjusts node heights to model changes in evolutionary rates over time, with δ < 1 indicating deceleration and δ > 1 suggesting acceleration toward the present. Pagel’s δ captures how rapid or steady trait evolution is over time [51]. Finally, the κ transformation alters branch lengths to explore how evolutionary distance affects trait similarity, with κ = 1 retaining the original tree structure and κ = 0 equalizing all branch lengths across the tree, representing a situation where change is proportional to the number of nodes separating species rather than time [40]. Pagel’s κ shows whether traits form gradually across lineages, or shift abruptly when new species form [51].

## METHODOLOGY

### Phylogenetic comparative methods currently utilized within comparative oncology

Given the diversity of phenotypic traits and the distinct data formats that they assume (e.g. discrete or continuous) there has been an equally diverse set of PCMs developed to analyze them. Given a specific phenotypic trait and the type of data collected, comparative phylogenetics has focused on identifying the evolutionary process that best explains how the trait has changed across the phylogeny. These methods are of interest in comparative oncology because they allow researchers to test both historical and contemporary questions about cancer in both humans and their phylogenetic relatives. For example, selecting the appropriate model of evolution can help researchers identify life-history traits that change in concert with cancer prevalence. However, there is debate about which methods are most appropriate for identifying best-fit models. For any discipline that is eager to integrate the use of PCMs, it can be daunting to face these debates and determine which method is best suited for their specific purpose. Across the field, a standard for choosing appropriate models and testing relationships has yet to be set. To illustrate the importance of choosing the most appropriate statistical model for given data and phylogeny, a simulation study was conducted. In our simulation, we compared two methods used in previous studies alongside our newly created *PGLSSEyOptim* function. The first method, used in Compton et al.’s *Cancer Prevalence Across Vertebrates* is the *PGLSSEyPagel* function [35]. The other method is the *Compar.gee* function from *ape.* The Compar.gee function, adapted from the approach described by Paradis and Claude (2002), employs a generalized estimation equation alongside a phylogenetic correlation structure in order to generate log-odd relationships [52]. This method does not use a continuous variable, such as species’ level cancer prevalence, but relies upon the occurrences and non-occurrences of the independent variable within each species. This method is used within Bulls et al.’s similar analysis of cancer across tetrapods to perform a binomial regression [34]**.** These models have distinct assumptions and characteristics, in which the researcher must take into account before deciding on which model to utilize.

### Aggregating necropsy data and integrating phylogenetic relatedness for species level analysis

To illustrate the utility of PCMs in cross-species analysis, we utilized a previously published dataset of cross-species cancer prevalence, containing data on 292 species and 16 049 individuals [35]. In short, the database is built on the necropsy records of each individual, collected, with permission, from 99 zoological institutions, aquariums, and animal housing facilities. The individual necropsy data was then aggregated, where relevant variables such as benign and malignant tumor incidence were summarized to represent each species. Species level data also includes the number of necropsy records and taxonomic information. The summarized necropsy data was then paired with species-specific adult mass data. Life history trait data was collected from two of the popular life history databases often utilized in comparative oncology studies: PanTHERIA and Amniote [53–55]. The phylogenetic tree for these species was obtained from TimeTree 5, which measures branch lengths in million years ago (Mya) [56].

### Fitting popular models of evolution to cross-species cancer prevalence data

To evaluate how a singular trait fits different evolutionary models, we calculated the probability of observing the data under each model using Maximum Likelihood Estimation, which identifies the parameter values that maximize the likelihood of the observed data. In Fig. 3, the focal trait was cancer prevalence; however, this process can be applied to any continuous, species-level variable. Maximum likelihood estimation is a well-established model-fitting method, with well-understood properties [57]. To compare models fairly, we used the Akaike Information Criterion (AIC), derived from the maximum likelihood framework, which adjusts model likelihoods by penalizing additional parameters that could lead to overfitting. Lower AIC values indicate relatively stronger support for the model. We implemented this approach using the fitContinuous function from the ***geiger*** R package [43]. These estimated parameters can then be applied to the most-likely evolutionary model to generate ancestral states of species-level traits that are consistent with the fitted model and its underlying assumptions.

In the results that follow, we focus on three PCMs for cancer oncology: first, identifying the best model of trait evolution; second, reconstructing ancestral character states; and third, testing for evolutionary correlations among traits. This workflow provides a systematic approach for incorporating ancestral history into statistical analysis. It establishes a foundation for testing evolution-informed hypotheses, such as the evolutionary mismatch framework, which often focuses on identifying correlates of cancer resistance across species. The outcomes of these analyses can then inform subsequent, more specialized methods tailored to specific hypotheses [58].

## RESULTS

### Variations in best fit evolutionary model

Across the tree of life, variations in life history strategies have led to a wide range of evolutionary solutions for managing disease risk. Rather than assuming a single, uniform mode of evolution, we used geiger’s fitContinous function to compare alternative models of trait evolution across taxonomic groups. Each model was fit using maximum likelihood, and model selection was based on AIC. A model with an AIC at least 10 units lower than competing models was considered to have substantially better support. However, when multiple models fell within 10 AIC units of one another, no single model was strongly favored—indicating model uncertainty [51]. In such cases, we assigned the best-fitting model from the higher taxonomic rank to maintain consistency in evolutionary assumptions. This approach ensures that only groups showing a clear improvement in model fit are interpreted as deviating from ancestral evolutionary dynamics. Determining the best fit model and its parameters, for a group of species, enables trait reconstruction that is reflective of the most-likely evolutionary path.

#### Reconstructing disease traits

Researchers can utilize the earlier discussed model fitting process to reconstruct disease trait data under the most-likely evolutionary model. In the case of the mammal data, Pagel’s λ model is the best fit. Ancestral states and confidence intervals for this model were estimated via the anc.ML function from *phytools*. Estimated ancestral states under Pagel’s model are represented by the white line, illustrating the inferred malignancy prevalence at internal nodes of the phylogeny. The surrounding blue haze represents the 95% confidence interval, capturing the range of uncertainty in these reconstructions and highlighting variability in trait evolution across mammalian lineages.

#### Adapting phylogenetic generalized least squares

Models and their fitted parameters can be utilized within the generalized least squares regression model. The method, *PGLSSeyPagel*, used in Compton et al. to test for statistical relationships between life history and cancer data, assumed Brownian motion [35]. With this, we developed a new function, *PGLSSeyOptim*. This method builds upon the *PGLS.SEy*, from phytools [59], and the *PGLSSeyPagel* method created by Diego Mallo. This function adds the ability to assume Brownian Motion, OU, Pagel’s lambda, delta, kappa, white noise, and rate trend. Parameter estimates within the *PGLSSeyOptim* utilize fitContinuous to ensure continuity between the fitting and the slope estimation process.

#### Comparing popular comparative methods in comparative oncology

Recent attempts to apply PCMs to cross-species disease prevalence data have utilized several statistical methods for phylogenetically informed regression. To assess the difference in these methods, we performed a simulation study aimed to show how evolutionary model fitting and statistical method selection affect statistical outcomes. The study used previously collected life history data from PanTHERIA and Amniote along with simulated cancer prevalence data, and simulated numbers of necropsies (i.e. sample size) to compare error rates between the different phylogenetic statistical methods. [53–55]. The methods analyzed in this simulation study came from Compton et al. ‘pgls.SEy’ [35], Bulls et al. ‘compare.gee’ [34] and an updated version of pgls.SEy; ‘pgls.SEy.Optim’. The simulation has two changing parameters, which are the minimum number of necropsies per species and the degree of the relationship (slope) between the life history predictor variable and the simulated neoplasia prevalence values. For each of these combinations, with each statistical method, 1000 iterations were completed before moving on to the next combination of parameters. Each iteration of the simulation first randomly samples the needed amount of species from the dataset. From there, the number of records is simulated using the negative binomial distribution. The negative binomial distribution most similarly mirrored the real distribution of necropsies per species from recent comparative oncology papers [18, 32, 34, 35]. The phylogenetic trees are then pruned to match the simulated data and standard errors are then calculated for each of the PGLS functions assessed. The relationships between simulated life history traits and cancer prevalence were generated using a simple linear slope-intercept function, with adjustments applied to reflect the evolutionary model under which the data were simulated. To prevent unrealistically perfect linear relationships and better approximate biological variability, we added a small random noise term drawn from a uniform distribution between −0.05 and 0.05. For the null noise simulations, both predictor and response variables were independently generated from uniform random distributions, representing the null hypothesis that no true relationship exists between traits. For compar.gee, neoplasia occurrences and non-occurrences were generated from the same data used for the Phylogenetic Generalized Least Squares (PGLS) tests.

Within the simulation study, three different models of evolution, OU, BM, and Pagel’s Lambda, were used to simulate trait data with a slope. The slope was randomly chosen for each trial between −2 and 2. For the random noise experiment, both independent and dependent variables were generated randomly. The number of records per species was chosen randomly for each species from the negative binomial distribution with a minimum threshold of 1, 5, 10 or 20 records per species, to mimic different studies in the literature [18, 32, 33, 35]. For the experiments with a given slope and model, a false negative error was indicated if the *P*-value was > 0.05. For the null experiment, false positive errors were indicated if the p-value was < 0.05. Across all parameter values and methods, the average error rates were 15.9% for the compar.gee binomial, 6.9% for PGLSSey, and 5.84% for PGLSSey.Optim. Additional summary statistics are in supplementary Table 2.

## CONCLUSIONS AND IMPLICATIONS

### Modes of evolution between clades

**Figure 1:**
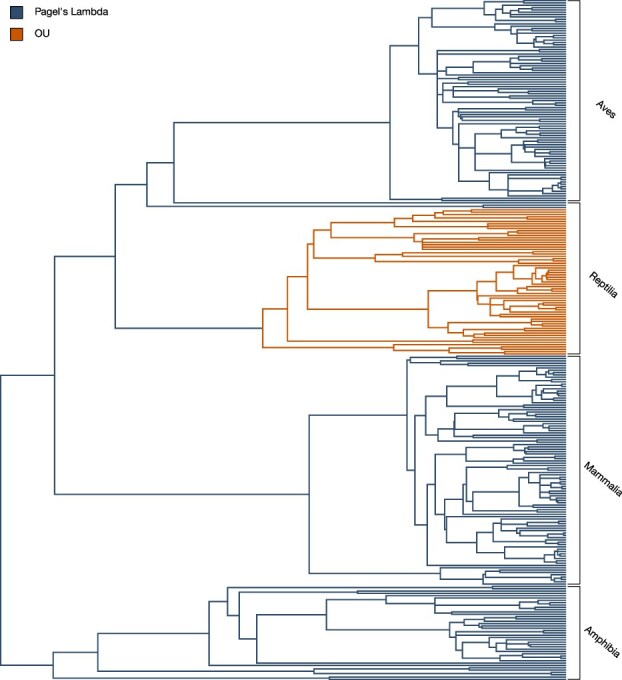
One phylogeny, multiple modes of evolution.

Figure 1 illustrates how the best-fit evolutionary models can vary across different branches of a phylogeny. While a single model may explain trait evolution over time across a large group of species, closer examination of specific clades reveals shifts in these models. Under the life history framework, natural selection leads species to adopt different trade-offs to optimize survival and reproduction. As species diverge and follow distinct life history strategies, their evolutionary trajectories can vary as a result. These changes in trait evolution often reflect shifts in those evolutionary trajectories, as species adapt to different ecological pressures and life history demands [21, 60–62]. Applying a single model across hundreds of species may fail to capture the diverse dynamics of trait evolution, especially when these species experience different evolutionary pressures. Utilizing multiple evolutionary models better reflects the varying rates and processes of evolution within clades.

Additionally, for a subgroup’s evolutionary model to be considered valid, it must exhibit a substantially better fit than competing models. On top of all of this, researchers must evaluate whether the biological characteristics of a trait align with the dynamics of the chosen evolutionary model. For example, if a trait is known to follow an evolutionary pattern consistent with Brownian motion, but a clade shows a marginally better fit for another model, researchers should assess whether or not that model is a reasonable hypothesis for the evolutionary dynamics of that trait. This approach provides a more accurate depiction of the complex, non-uniform evolutionary paths that shape adaptations, including those related to disease prevention, while keeping findings grounded in biological reality. As Beaulieu et al. (2012) argue, biological reality is too complex for any single model to fully capture, and the fit of a model depends on both its parameters and the available data [63]. Using multiple models helps account for the varied evolutionary pressures across species.

### Insights from ancestral state reconstruction

**Figure 2:**
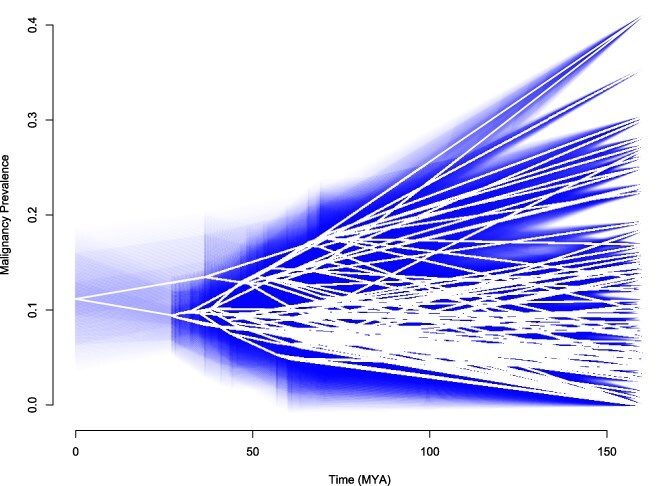
Ancestral state reconstruction of malignancy prevalence under Pagel’s lambda model over time for 98 mammalian species.

Understanding the evolutionary history of cancer prevalence across species has significant implications for human medicine. In Fig. 2, we reconstruct cancer prevalence across a group of mammals, using Pagel’s λ model, which was the best-fit model. Evolutionary biologists can utilize ancestral state reconstruction under a best-fit model of evolution to infer how disease traits have changed over time across lineages. If specific genetic variations or evolutionary pressures are associated with higher or lower cancer risk in certain lineages, these findings can guide human cancer research by highlighting conserved or divergent biological pathways involved in tumor suppression, DNA repair, or immune response [64–68]. Ancestral state reconstructions can be applied beyond cancer research, providing valuable insights into the evolutionary drivers of various diseases that impact human health.

### Enhancing phylogenetic analyses by accounting for evolutionary dynamics

**Figure 3:**
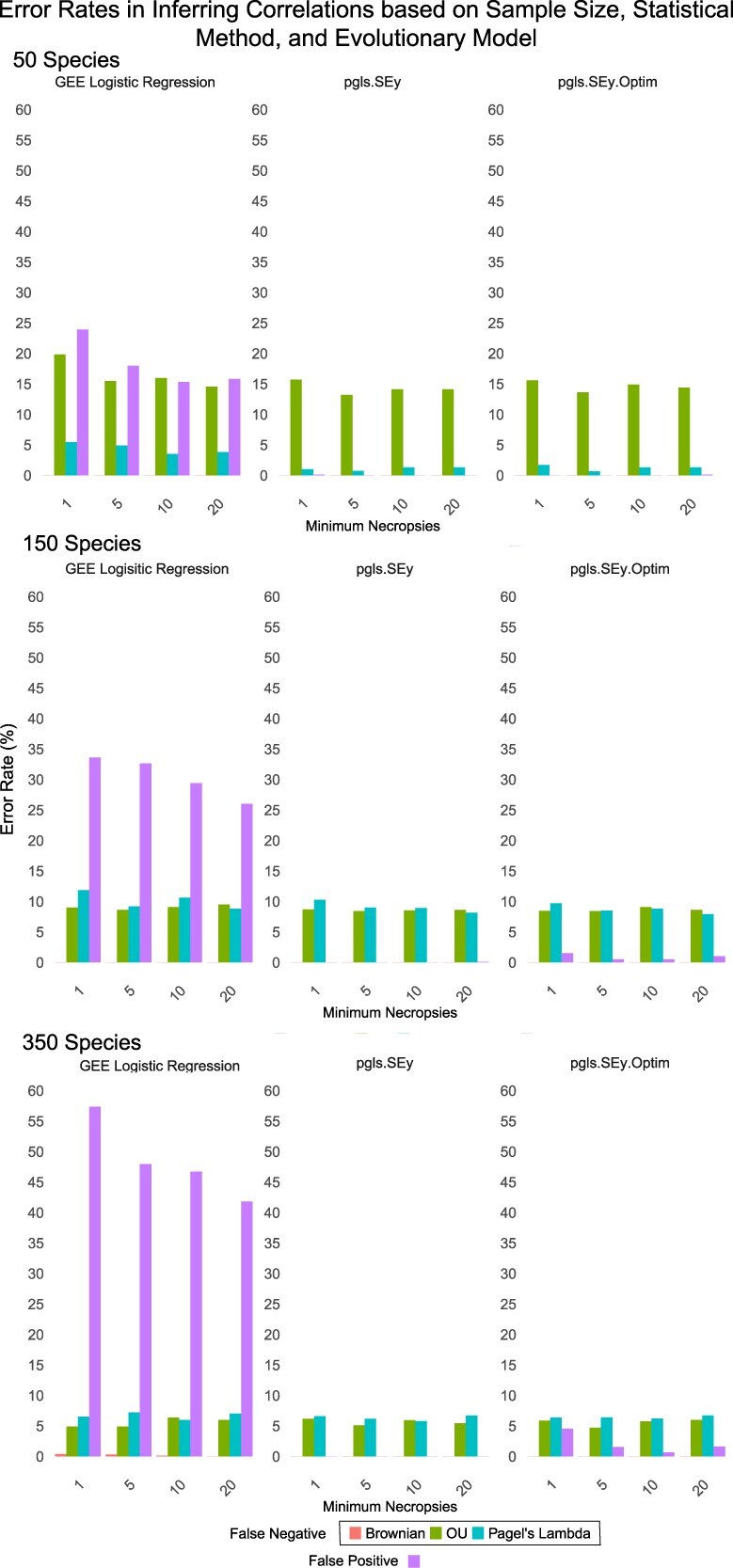
Simulation results for minimums of 1,5,10, and 20 records for 50, 150 or 350 random mammalian species. False positive error rates were based on simulations with no relationship between the independent and dependent variables, representing the null hypothesis of no true statistical relationship. False negative error rates were calculated based on simulated data that had a linear relationship between the independent and dependent variables with a slope randomly chosen uniformly between −2 and 2, but that changed along the phylogeny based on one of three models: Brownian motion (Brownian), Ornstein-Uhlenbeck (OU) or Pagel’s lambda.

Accurate estimates of the relationships between traits depend not only on the regression method but also on the evolutionary model assumed during analysis. Fitting evolutionary models allows researchers to infer the most likely evolutionary history of the trait being studied. This approach is similar to that of Brocklehurst in 2016, who similarly tested for the best fit evolutionary model to analyze evolution rates and variations of body size [69]. Our analyses demonstrate that incorporating well-fitted evolutionary models and their estimated parameters can reduce overall error rates in phylogenetic comparative methods (Fig. 3).

To support this workflow, we adapted PGLS.SEy from the phytools package to create PGLSeyOptim, a tool that streamlines evolutionary model selection, parameter fitting, and regression estimation. As phylogenetic comparative methods become increasingly central to evolutionary medicine, it is important to recognize that different traits—and different phylogenies—may evolve under distinct evolutionary processes. Disease-related traits, in particular, may follow dynamics that differ from those of morphological or ecological traits. Failing to account for these differences can lead to inaccurate estimates of trait correlations and misleading evolutionary interpretations. Researchers should consider not only statistical fit, but also whether a model’s evolutionary assumptions make biological sense in the context of the trait being studied. Tools like PGLSeyOptim help facilitate this process by enabling researchers to identify and apply the best-fitting evolutionary model for their data before conducting phylogenetic regression.

### Model assumptions in phylogenetic comparative methods

Across all simulations, the regression methods exhibited similar false negative rates regardless of mode of evolution, number of species, or number of observations per species (Fig. 3, ST2). This suggests that these methods appear to be well suited for analyzing disease susceptibility if it evolved randomly over evolutionary time (Brownian motion). That is, there were very few instances of false negative errors when we simulated the trait evolving via Brownian motion, regardless of the type of regression used. However, when disease susceptibility evolved under the OU model all regression methods were susceptible to some level of false negatives, which was worse when there were fewer species in the analyses.

Our results highlight how violating model assumptions can distort statistical inference in cross-species trait analysis. Both PGLS versions performed consistently across all scenarios, because the data that is simulated matches the assumption that the continuous GLS models assume (Fig. 3). In the case of the logistic regression, we saw higher rates of false positives across all simulations. The method used does not account for overdispersion or variation in trails (e.g. necropsy records), which are common in zoological datasets. This results in underestimated standard errors and inflated significance.

The misalignment between data and the assumptions of the logistical model was intentional. We designed the simulation to highlight the consequences of applying a method without verifying its assumptions, as well as assessing the robustness of these methods to violations of assumptions that might be common in cancer prevalence data. Our aim is not to discredit the phylogenetic logistic regression, but to show how using an inappropriate statistical approach for the types of data common in comparative studies can lead to skewed or misleading results. In fact, there are several PCMs that utilize logistic regressions and count data that can account for overdispersion and additional distribution families such as the MCMCglmm R package [70].

Other groups have employed regressions using phylogenetic independent contrasts instead of phylogenetic variance covariance matrices to mitigate potential errors in regression estimates caused by phylogenetic correlations. This approach is particularly useful when researchers aim to minimize the influence of phylogenetic signal and evolutionary history in their analyses [71]. However, when studying evolving disease traits in a comparative setting, incorporating phylogenetic relationships is crucial for understanding trends within an evolutionary context.

Comparative phylogenetics has a clear, complementary place alongside comparative genomics in explaining the origin, risk, and persistence of human pathologies. As comparative data on diseases in non-human species increases, so shall the need for the robust utilization of comparative phylogenetic methods. Like many other subfields within evolutionary medicine, comparative oncology is a nascent field and we should expect to see an array of methodological approaches to other diseases as well (e.g. studying cardiovascular disease across the tree of life). However, just as genomics has managed to do, there must be agreed upon ‘best practices’ for the analyses of disease prevalence data. While these best practices must be an emergent property of peer-review and discussion amongst the field, we hope that we have at least laid the groundwork for how to evaluate the robustness of these methods within evolutionary medicine.

Evolutionary medicine has made important insights into the origin and persistence of human disease. As the discipline has diversified, it has continued to permeate new fields of disease and demonstrated its broad utility to medicine. Despite its successes, much of the progress made in evolutionary medicine has been conceptual, expanding our understanding of human disease within the already established framework of what we know about humans’ evolutionary history. Primary explorations of human-relevant disease are possible through the classical methods employed in evolutionary biology, namely comparative phylogenetics. When we compare human diseases across species the prevalence of that disease is well understood as a continuous species trait. Virtually every human disease that can be viewed through the lens of epidemiology (i.e. a measurement of its prevalence in a population) can be studied in a similar way across species through comparative phylogenetics. As comparative phylogenetics becomes increasingly integrated into disease research, it is essential to establish best practices to ensure accurate and meaningful results that will significantly contribute to our understanding of cancer.

## Supplementary Material

ST1ModelFit_eoaf039

ST2errorRates_eoaf039

## Notes

‘We must learn to treat comparative data with the same respect as we would experimental results.’ (Maynard Smith and Holliday 1979, p. vii)

## References

[1] Nesse RM, Williams GC. Evolution and the origins of disease. *Sci Am* 1998;279:86–93. 9796548 10.1038/scientificamerican1198-86

[2] Williams GC, Nesse RM. The dawn of Darwinian medicine. *Q Rev Biol* 1991;66:1–22. 2052670 10.1086/417048

[3] Nesse RM, Williams GC. On Darwinian Medicine [Internet]. www-personal.umich.edu; 1999 [cited 2023 May 30]. University of Michigan. Available from: http://www-personal.umich.edu/∼nesse/Articles/OnDarMed-LifeScience-1999.PDF

[4] Lea AJ, Clark AG, Dahl AW et al. Evolutionary Mismatch and the Role of GxE Interactions in Human Disease. ArXiv [Internet]. 2023 Feb 13; medrxiv. Available from: 10.1101/2021.10.24.21265442

[5] Manus MB . Evolutionary mismatch. *Evol Med Public Health* 2018;2018:190–1. https://doi.org/10.1093/emph/eoy023 30159142 10.1093/emph/eoy023 PMC6109377

[6] Lea AJ, Clark AG, Dahl AW et al. Applying an evolutionary mismatch framework to understand disease susceptibility. *PLoS Biol* 2023;21:e3002311. https://doi.org/10.1371/journal.pbio.3002311 37695771 10.1371/journal.pbio.3002311 PMC10513379

[7] Bourrat P, Griffiths PE. The idea of mismatch in evolutionary medicine. *Br J Philos Sci [Internet]* 2021;17. Jul 20; Available from: 10.1086/716543

[8] Turaman C . Classification of the risk factors of coronary heart disease and their evolutionary origins. *Health Sci Rev* 2022;3:100027.

[9] Keestra SM, Male V, Salali GD. Out of balance: The role of evolutionary mismatches in the sex disparity in autoimmune disease. *Med Hypotheses* 2021;151:110558. 33964604 10.1016/j.mehy.2021.110558

[10] Campbell BC, Cajigal A. Diabetes: Energetics, development and human evolution. *Med Hypotheses* 2001;57:64–7. 11421628 10.1054/mehy.2001.1309

[11] Nesse RM . Fear and fitness: An evolutionary analysis of anxiety disorders. *Ethol Sociobiol [Internet]* 1994;15:247–261. Available from: https://www.sciencedirect.com/science/article/pii/0162309594900027

[12] Nesse RM . Good Reasons for Bad Feelings: Insights from the Frontier of Evolutionary Psychiatry. New York: Penguin Press; 2019. Available from: https://books.google.ca/books?hl=en&lr=&id=frmHDwAAQBAJ&oi=fnd&pg=PR13&ots=TBHFiHMFwl&sig=4usb1AswcmAYa-x4Ex8z_v5i9m8

[13] Nesse RM . Why Evolutionary Do Mental Disorders Persist? Perspectives on Evolution and Mental Health. Cambridge: Cambridge University Press; 2022. Available from: https://books.google.com/books?hl=en&lr=&id=Tph-EAAAQBAJ&oi=fnd&pg=PA84&dq=Why+Evolutionary+Mental+Disorders+Persist&ots=d6KaREPMi8&sig=bnsD-2uUjZKVB6BjEd76YEgWgB0

[14] Nesse RM . Is depression an adaptation? *Arch Gen Psychiatry [Internet]* 2000;57:14–20. Available from: https://jamanetwork.com/journals/jamapsychiatry/article-abstract/481547 10.1001/archpsyc.57.1.14 10632228

[15] Cheng A, Jaint D, Thomas S et al. Overcoming evolutionary mismatch by self-treatment with helminths: Current practices and experience. *J Evol Med* 2015;2015:1–22.

[16] Jacqueline C, Biro PA, Beckmann C et al. Cancer: A disease at the crossroads of trade-offs. *Evol Appl* 2017;10:215–25. https://doi.org/10.1111/eva.12444 28250806 10.1111/eva.12444 PMC5322410

[17] Nedelcu AM . Evo-devo perspectives on cancer. *Essays Biochem* 2022;66:797–815. https://doi.org/10.1042/EBC20220041 36250956 10.1042/EBC20220041

[18] Kapsetaki SE, Compton ZT, Dolan J et al. Life history traits and cancer prevalence in birds. *Evol Med Public Health [Internet]* 2024; 12(1):105–116. Jun 27; Available from: 10.1093/emph/eoae011/7700206 PMC11297545 39099847

[19] Boddy AM, Abegglen LM, Pessier AP et al. Lifetime cancer prevalence and life history traits in mammals. *Evol Med Public Health* 2020;2020:187–95. https://doi.org/10.1093/emph/eoaa015 33209304 10.1093/emph/eoaa015 PMC7652303

[20] Dujon AM, Boutry J, Tissot S et al. Cancer susceptibility as a cost of reproduction and contributor to life history evolution. *Front Ecol Evol [Internet]* 2022;10:861103. Available from: 10.3389/fevo.2022.861103

[21] Brommer JE . The evolution of fitness in life-history theory. *Biol Rev Camb Philos Soc* 2000;75:377–404. 11034016 10.1017/s000632310000551x

[22] Boddy AM, Kokko H, Breden F et al. Cancer susceptibility and reproductive trade-offs: A model of the evolution of cancer defences. *Philos Trans R Soc Lond B Biol Sci [Internet]* 2015;370:20140220. https://doi.org/10.1098/rstb.2014.0220 26056364 10.1098/rstb.2014.0220 PMC4581025

[23] Boddy AM, Harrison TM, Abegglen LM. Comparative oncology: New insights into an ancient disease. *iScience* 2020;23:101373. https://doi.org/10.1016/j.isci.2020.101373 32738614 10.1016/j.isci.2020.101373 PMC7394918

[24] Cornwallis CK, Griffin AS. A guided tour of phylogenetic comparative methods for studying trait evolution. *Annu Rev Ecol Evol Syst [Internet]* 2024;55:181–204. Aug 6 [cited 2024 Oct 22]; Available from: 10.1146/annurev-ecolsys-102221-050754

[25] Felsenstein J . Phylogenies and the comparative method. *Am Nat* 1985;125:1–15. 10.1086/703055 31094602

[26] Felsenstein J . Phylogenies from molecular sequences: Inference and reliability. *Annu Rev Genet* 1988;22:521–65. 3071258 10.1146/annurev.ge.22.120188.002513

[27] Felsenstein J . Phylogenies and quantitative characters. *Annu Rev Ecol Syst [Internet]* 1988;19:445–571. Available from: https://www.jstor.org/stable/2097162?casa_token=MpYB7MCAtCsAAAAA:1svZfnl3lAF8SXNE4FVQFUUnpf-2QAHzvJkzBzcHgYupr7plC1eAcL7hHyl3NpZlhlpOjeViNczJq8ob7efuE3OExcZdcHdTvBlO23l9GJw0c8GMQg

[28] Pagel MD, Harvey PH. Comparative methods for examining adaptation depend on evolutionary models. *Folia Primatol (Basel)* 1989;53:203–20. 2691365 10.1159/000156417

[29] Ridley M . The Explanation of Organic Diversity: The Comparative Method and Adaptations for Mating. New York: Oxford University Press, 1983. 1 online resource (viii, 272 pages illustrations). (Oxford science publications).

[30] Garland T Jr, Bennett AF, Rezende EL. Phylogenetic approaches in comparative physiology. *J Exp Biol* 2005;208:3015–35. 16081601 10.1242/jeb.01745

[31] Rezende EL, Diniz-Filho JAF. Phylogenetic analyses: Comparing species to infer adaptations and physiological mechanisms. *Compr Physiol* 2012;2:639–74. 23728983 10.1002/cphy.c100079

[32] Vincze O, Colchero F, Lemaître JF et al. Cancer risk across mammals. *Nature.* 2022;601:263–7. https://doi.org/10.1038/s41586-021-04224-5 34937938 10.1038/s41586-021-04224-5 PMC8755536

[33] Boddy AM, Abegglen LM, Pessier AP et al. Lifetime cancer prevalence and life history traits in mammals. *Evol Med Public Health [Internet]* 2020;2020:187–195. May 25 [cited 2020 Jul 9]; Available from: 10.1093/emph/eoaa015/33293561/eoaa015.pdf PMC7652303 33209304

[34] Bulls SE, Platner L, Ayub W et al. Cancer Prevalence Is Related to Body Mass and Lifespan in Tetrapods and Remarkably Low in Turtles [Internet]. bioRxiv 2023. [cited 2023 Mar 21]. p. 2022.07.12.499088. biorxiv. Available from: https://www.biorxiv.org/content/biorxiv/early/2023/02/24/2022.07.12.499088

[35] Compton ZT, Mellon W, Harris VK et al. Cancer prevalence across vertebrates. *Cancer Discov* 2025;15:227–244. Available from: https://aacrjournals.org/cancerdiscovery/article/15/1/227/750844/Cancer-Prevalence-across-VertebratesCancer-across 10.1158/2159-8290.CD-24-0573 PMC11726020 39445720

[36] McShea DW, Wang SC, Brandon RN. A quantitative formulation of biology’s first law. *Evolution.* 2019;73:1101–15. https://doi.org/10.1111/evo.13735 30980538 10.1111/evo.13735

[37] Cornwell W, Nakagawa S. Phylogenetic comparative methods. *Curr Biol* 2017;27:R333–6. https://doi.org/10.1016/j.cub.2017.03.049 28486113 10.1016/j.cub.2017.03.049

[38] Castiglione S, Tesone G, Piccolo M et al. A new method for testing evolutionary rate variation and shifts in phenotypic evolution. *Methods Ecol Evol* 2018;9:974–83.

[39] Revell LJ, Harmon LJ, Collar DC. Phylogenetic signal, evolutionary process, and rate. *Syst Biol* 2008;57:591–601. https://doi.org/10.1080/10635150802302427 18709597 10.1080/10635150802302427

[40] Harmon LJ . Phylogenetic Comparative Methods, 2019.Independently Published Available from: https://ecoevorxiv.org/repository/object/4486/download/9000/?embed=True

[41] Hunt G, Hopkins MJ, Lidgard S. Simple versus complex models of trait evolution and stasis as a response to environmental change. *Proc Natl Acad Sci USA* 2015;112:4885–90. https://doi.org/10.1073/pnas.1403662111 25901309 10.1073/pnas.1403662111 PMC4413263

[42] Harmon LJ, Weir JT, Brock CD et al. GEIGER: Investigating evolutionary radiations. *Bioinformatics.* 2008;24:129–31. 18006550 10.1093/bioinformatics/btm538

[43] Pennell MW, Eastman JM, Slater GJ et al. Geiger v2.0: An expanded suite of methods for fitting macroevolutionary models to phylogenetic trees. *Bioinformatics.* 2014;30:2216–8. https://doi.org/10.1093/bioinformatics/btu181 24728855 10.1093/bioinformatics/btu181

[44] Revell LJ . Ancestral character estimation under the threshold model from quantitative genetics. *Evolution.* 2014;68:743–59. https://doi.org/10.1111/evo.12300 24152239 10.1111/evo.12300

[45] Baker J, Meade A, Pagel M et al. Adaptive evolution toward larger size in mammals. *Proc Natl Acad Sci USA* 2015;112:5093–8. https://doi.org/10.1073/pnas.1419823112 25848031 10.1073/pnas.1419823112 PMC4413265

[46] Uhlenbeck GE, Ornstein LS. On the theory of the Brownian motion. *Phys Rev [Internet]* 1930;36:823. Available from: 10.1103/PhysRev.36.823

[47] Felsenstein J . Maximum-likelihood estimation of evolutionary trees from continuous characters. *Am J Hum Genet* 1973;25:471–92. 4741844 PMC1762641

[48] Blomberg SP, Rathnayake SI, Moreau CM. Beyond Brownian motion and the Ornstein-Uhlenbeck process: Stochastic diffusion models for the evolution of quantitative characters. *Am Nat* 2020;195:145–65. https://doi.org/10.1086/706339 32017624 10.1086/706339

[49] Hunt G . Measuring rates of phenotypic evolution and the inseparability of tempo and mode. *Paleobiology.* 2012;38:351–73.

[50] Pagel M . Inferring the historical patterns of biological evolution. *Nature.* 1999;401:877–84. 10553904 10.1038/44766

[51] Burnham KP, Anderson DR. Multimodel inference: Understanding AIC and BIC in model selection. *Sociol Methods Res* 2004;33:261–304.

[52] Paradis E, Claude J. Analysis of comparative data using generalized estimating equations. *J Theor Biol* 2002;218:175–85. 12381290 10.1006/jtbi.2002.3066

[53] Jones KE, Bielby J, Cardillo M et al. PanTHERIA: A species-level database of life history, ecology, and geography of extant and recently extinct mammals: Ecological archives E090-184. *Ecology.* 2009;90:2648.

[54] de Magalhães JP, Costa J. A database of vertebrate longevity records and their relation to other life-history traits. *J Evol Biol* 2009;22:1770–4. https://doi.org/10.1111/j.1420-9101.2009.01783.x 19522730 10.1111/j.1420-9101.2009.01783.x

[55] Myhrvold NP, Baldridge E, Chan B et al. An amniote life-history database to perform comparative analyses with birds, mammals, and reptiles. *Ecology.* 2015;96:3109–000.

[56] Kumar S, Suleski M, Craig JM et al. TimeTree 5: An expanded resource for species divergence times. *Mol Biol Evol* 2022;39:msac174. 35932227 10.1093/molbev/msac174 PMC9400175

[57] Rayner JCW, Best DJ. Smooth tests of goodness of fit: An overview. *Int Stat Rev* 1990;58:9.

[58] Revell LJ, Harmon LJ. Phylogenetic Comparative Methods in R, 2022. Princeton: Princeton University Press. Available from: https://books.google.com/books?hl=en&lr=&id=9qJjEAAAQBAJ&oi=fnd&pg=PP1&dq=revell+harmon+2022&ots=TNTlb6Bd0R&sig=b4mKHyYw_OdBh0cwryKPDvMc0bk.

[59] Revell LJ . Phytools: An R package for phylogenetic comparative biology (and other things). *Methods Ecol Evol* 2012;3:217–23.

[60] Stearns SC . Life history evolution: Successes, limitations, and prospects. *Naturwissenschaften.* 2000;87:476–86. 11151666 10.1007/s001140050763

[61] Lika K, Kooijman SALM. Life history implications of allocation to growth versus reproduction in dynamic energy budgets. *Bull Math Biol* 2003;65:809–34. 12909252 10.1016/S0092-8240(03)00039-9

[62] Roff D . In: Roff DA (ed.), *Evolution of Life Histories: Theory and Analysis*1993rd edn, p. 548. New York, NY: Springer, 1993.

[63] Beaulieu JM, Jhwueng DC, Boettiger C et al. Modeling stabilizing selection: Expanding the Ornstein-Uhlenbeck model of adaptive evolution. *Evolution.* 2012;66:2369–83. https://doi.org/10.1111/j.1558-5646.2012.01619.x 22834738 10.1111/j.1558-5646.2012.01619.x

[64] Greaves M . Evolutionary determinants of cancer. *Cancer Discov* 2015;5:806–20. https://doi.org/10.1158/2159-8290.CD-15-0439 26193902 10.1158/2159-8290.CD-15-0439 PMC4539576

[65] Abegglen LM, Caulin AF, Chan A et al. Potential mechanisms for cancer resistance in elephants and comparative cellular response to DNA damage in humans. *JAMA.* 2015;314:1850–60. https://doi.org/10.1001/jama.2015.13134 26447779 10.1001/jama.2015.13134 PMC4858328

[66] Vazquez JM, Pena MT, Muhammad B et al. Parallel evolution of reduced cancer risk and tumor suppressor duplications in Xenarthra. *Elife [Internet]* 2022;11:e82558. Available from: https://doi.org/10.7554/eLife.82558 10.7554/eLife.82558 PMC9810328 36480266

[67] Seluanov A, Gladyshev VN, Vijg J et al. Mechanisms of cancer resistance in long-lived mammals. *Nat Rev Cancer* 2018;18:433–41. https://doi.org/10.1038/s41568-018-0004-9 29622806 10.1038/s41568-018-0004-9 PMC6015544

[68] Vazquez JM, Sulak M, Chigurupati S et al. A zombie LIF gene in elephants is upregulated by TP53 to induce apoptosis in response to DNA damage. *Cell Rep* 2018;24:1765–76. https://doi.org/10.1016/j.celrep.2018.07.042 30110634 10.1016/j.celrep.2018.07.042

[69] Brocklehurst N . Rates and modes of body size evolution in early carnivores and herbivores: A case study from Captorhinidae. *PeerJ.* 2016;4:e1555. 26793424 10.7717/peerj.1555 PMC4715457

[70] Hadfield JD . MCMC methods for multi-response generalized linear mixed models: TheMCMCglmmRPackage. *J Stat Softw* 2010;33:1–22.20808728

[71] Adams R, Cain Z, Assis R et al. Robust phylogenetic regression. *Syst Biol* 2024;73:140–57. https://doi.org/10.1093/sysbio/syad070 38035624 10.1093/sysbio/syad070 PMC11129599

